# Identification of gene polymorphisms of human DNA topoisomerase I in the National Cancer Institute panel of human tumour cell lines

**DOI:** 10.1038/sj.bjc.6603361

**Published:** 2006-09-19

**Authors:** F Moisan, M Longy, J Robert, V Le Morvan

**Affiliations:** 1 Laboratoire de Pharmacologie des Agents Anticancéreux, Institut Bergonié, 229 Cours de l'Argonne, 33076, Bordeaux-cedex, France; 2 Université Victor Segalen Bordeaux 2, 146 rue Léo-Saignat, 33076, Bordeaux-cedex, France; 3 Laboratoire de Génétique, Institut Bergonié, 229 Cours de l'Argonne, 33076, Bordeaux-cedex, France

**Keywords:** anticancer drug cytotoxicity, camptothecins, DNA topoisomerase 1, gene polymorphisms, NCI-60 panel

## Abstract

Topoisomerase 1 (Top1), a nuclear enzyme involved in DNA relaxation, is the target of several anticancer drugs. *TOP1* mutations occur in camptothecin-resistant tumour cell lines. We explored, in the NCI panel of 60 human tumour cell lines, whether polymorphic variations in the *TOP1* gene could explain differences in drug sensitivity. The 21 exons of the gene were fully studied as well as five intronic domains that had previously been shown to harbour single nucleotide polymorphisms (SNPs) or mutations. PCR products covering the whole exonic sequences or the relevant intronic domains were subjected to denaturing high-performance liquid chromatography. Nucleotide variations were then determined by sequencing. Discrimination between intronic common and variant homozygous samples was performed using a restriction fragment length polymorphism technique. Only one exonic mutation was detected, at the heterozygous state; it occurs in exon 19 of a colon cancer cell line (HCT-15) and consists of a G>A transition at position 75, resulting in a Met675Ile change. The intronic sequences studied harboured the SNPs expected with allelic frequencies between 20 and 40%. Three major haplotypes, generating 92% of the 10 genotypes encountered, were defined as containing none of the intronic SNPs, or three of them, or all of them. No significant relationship was evidenced between Top1 expression and the *TOP1* polymorphisms studied. However, when comparing the cytotoxicity of 138 drugs as a function of the genotypes, several drug groups, namely Top1 inhibitors, antifolates and taxanes, had significantly different IC_50_s as a function of the distribution of the intronic SNPs of the *TOP1* gene.

Human DNA topoisomerase I (Top1) is a monomeric nuclear protein of 91 kDa (765 amino acids) encoded by a gene located on chromosome 20 (20q12–13.2). Topoisomerase 1 is the target enzyme of several anticancer drugs used in the clinics (camptothecins) or in development (indolocarbazoles) (Pommier *et al*, 1999). Topoisomerase 1 is able to relax supercoiled DNA, downstream the replication and transcription machineries, through the formation of transitory single-strand breaks. During the catalytic process, a transesterification occurs, with tyrosine 723 of the active centre forming a covalent bond with a 3′ phosphate group, allowing the nicked DNA strand to rotate about the intact strand, and relieving thus by one turn the torsional constraint. Topoisomerase 1 also catalyses the religation of nicked strand, restoring DNA continuity. Topoisomerase 1-interfering drugs convert the enzyme into a cellular poison by inhibiting the religation step, trapping thus the enzyme into a stable covalent DNA complex. Cytotoxic lesions are likely to result from the collision between the replication fork and the stabilised cleavable complex, transforming thus a single-strand break into a double-strand break (Wang, 1994). It should be noticed that some agents behave as catalytic inhibitors, by inhibiting the cleavage reaction itself; none of them has been yet developed as an anticancer agent (Pommier *et al*, 1998).

No functional analysis of the polymorphisms of the *TOP1* gene has been undertaken; such polymorphisms could lead to an alteration of the level of expression and/or the activity of the enzyme, which would explain part of the individual variability of response to Top1 inhibitors. Mutations of the *TOP1* gene have been observed in tumour cell lines selected for resistance to camptothecin (Takatani *et al*, 1997; Pommier *et al*, 1999; Tsurutani *et al*, 2002; Woo *et al*, 2002) or sometimes in clinical specimens (Ohashi *et al*, 1996), indicating that amino-acid changes could have consequences on cell response to this drug. In addition, a decreased expression of Top1 has been associated to resistance to camptothecin (Sorensen *et al*, 1995); although the mechanism involved in this decrease has not been identified, it could result from alterations of regulatory sequences in the promoter of the gene. Numerous functional polymorphisms have been identified in drug-metabolising enzymes or drug transporters, as well as in drug receptors and targets (see Robert *et al*, 2005, for review). For instance, a polymorphism located in the 5′ untranslated domain of thymidylate synthase, the target enzyme for fluoropyrimidines, is associated with a change in protein expression and, consequently, in cellular response to the drug (Pullarkat *et al*, 2001). Therefore, identifying functional polymorphisms in the *TOP1* gene could prove of interest for understanding the individual variability of drug effects.

Research and identification of functional polymorphisms in genes involved in drug response and toxicity generally require abundant clinical resources, with numerous patients treated with the drug and fully documented clinical files. This is especially difficult in oncology, because patients are often treated with drug combinations and because the clinical end points are multiple and sometimes difficult to record. Looking for a cellular model able to provide clues and tracks about the functional polymorphisms of the *TOP1* gene, we decided to use the National Cancer Institute (NCI) panel of 60 human tumour cell lines. This panel was initially established for high-throughput screening of natural products and synthetic molecules, on the basis of their antiproliferative properties (Monks *et al*, 1991). In addition, a number of molecular markers and gene expression profiles have been determined in the panel, allowing to establish relationships between chemo-sensitivity or -resistance and the molecular features of the cells (Scherf *et al*, 2000). The inverse correlation between fluorouracil cytotoxicity and dihydropyrimidine dehydrogenase gene expression has served as a paradigm for such studies. We and others have developed data mining on the NCI database in order to discover relationships between drug activity and cell molecular properties (Robert *et al*, 2004; Vekris *et al*, 2004). It should be mentioned that no relationship between *TOP1* gene expression and camptothecin cytotoxicity has been detected (Goldwasser *et al*, 1995).

No analysis of gene polymorphisms has yet been performed on the NCI panel. As it consists of tumour cells, they can present many somatic genetic alterations, distinct from an actual constitutive polymorphism present in the patient who hosted the tumour. We think, however, that the NCI panel could represent a valuable starting point to study the role of gene point variations on anticancer drug activity. The *TOP1* gene covers about 100 kb and consists of 21 exons; 248 single nucleotide polymorphisms (SNPs) have been referenced in databases (http://www.ncbi.nlm.nih.gov/SNP/snp_ref.cgi?locusId=7150), but the frequency of only 49 has been established. One of them only is present in a coding domain (exon 12), with a heterozygosity frequency of 0.043 in an African-American population but it was not detected in a Caucasian population (http://www.ncbi.nih.gov/SNP/snp_ss.cgi?subsnp_id=23535865). In a first step, we explored in totality the 21 exons of the gene and the 5′ untranscribed region by denaturing high-performance chromatography. In a second step, we analysed specifically five intronic SNPs chosen among the 15 that were known to have a frequency higher than 0.25, localised in introns 2, 3, 6, 8 and 17, covering about 82 kb. In addition to the study of the NCI panel, we also explored the *TOP1* exons of a standard Caucasian population. The relationship between the presence of a given SNP in the *TOP1* gene of the NCI panel cell lines and the expression of the *TOP1* gene or the cytotoxicity of 138 standard anticancer drugs was then studied, based on the publicly available NCI databases (http://dtp.nci.nih.gov).

## MATERIALS AND METHODS

### Biological samples

Frozen cell pellets from 59 of the 60 NCI cell lines of the panel were kindly provided by Dr S Holbeck, Cancer Therapeutic Branch, NCI, Bethesda, MD, USA. One cell line, MDA-N, is no longer available in the panel.

Genomic DNAs of a healthy Caucasian French population (53 samples) were kindly provided by the Laboratory of Genetics of Institut Bergonié. All individuals had given written consent for a scientific use of their blood samples.

### Molecular biology techniques

Genomic DNA was extracted from cell pellets using QIAamp® DNA minikit from Qiagen (Courtaboeuf, France). It was quantified by spectrophotometry. DNA electrophoresis was performed on agarose gels in Tris-acetate-EDTA buffer (pH 8.0).

**Figure 1:**
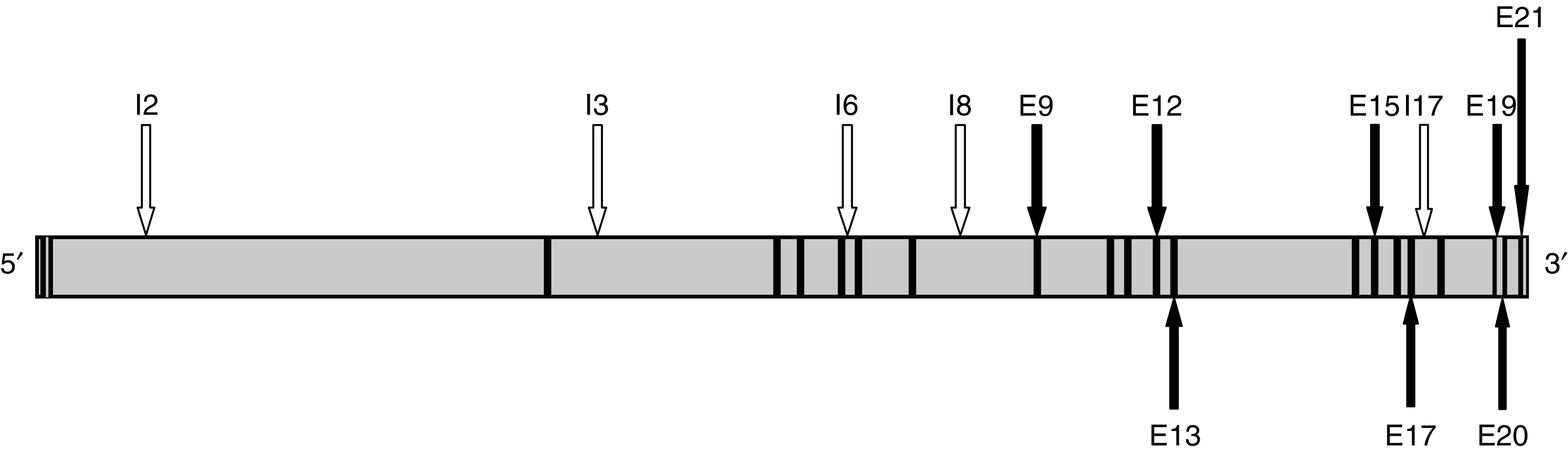
Genomic structure of the *TOP1* gene. The exons (E) and introns (I) specifically mentioned in the text are indicated by arrows and they are numbered accordingly.

Polymerase chain reactions (PCR) were performed on 40 ng genomic DNA, using Platinum® Taq polymerase (Invitrogen, Cergy-Pontoise, France) and a GenAmp PCR system 9700® thermocycler (Applied Biosystems, Courtaboeuf, France). The oligonucleotide primers were determined using the Primer 3 software, from the *TOP1* gene sequence (NT_011362). They were designed in order to cover the complete sequences of the exons, the 5′ untranscribed region up to 350 nucleotides upstream exon 1, and, for introns, the short sequences where a SNP had been localised (see Figure 1). Tables 1 and 2 present the sequences of the primers used for PCR as well as the length of the PCR products.

PCR products were submitted to denaturing high-performance liquid chromatography (dHPLC) in order to identify heterozygous DNA samples in the *TOP1* exons of the cell lines of the panel. We used a Wave Nucleic Acid Fragment Analysis System® (Transgenomic, Elancourt, France) with a ion-exchange column and a gradient of acetonitrile in triethyl ammonium acetate buffer as a solvent. In this system, heteroduplexes are eluted more rapidly than homoduplexes, revealing the presence of a SNP on one DNA strand. Homozygous variants cannot be discriminated from homozygous common alleles with this system.

Nucleotide sequencing of PCR products was achieved using an ABI 377 sequencer (Perkin Elmer, Courtaboeuf, France) and the ABI PRISM Dye Terminator Cycle Sequencing® kit (Perkin Elmer), which uses the dideoxynucleotide technique of Sanger *et al* (1977) with four different fluorochromes.

A restriction fragment length polymorphism (RFLP) technique could be set up for four of the five known intronic SNPs, using the same PCR primers as those used for dHPLC. This allowed the rapid identification of homozygous (common and variant) samples for each of the variations, as well as a confirmation for the heterozygous samples. Table 2 lists the restriction enzymes that were used for the digestions. All of them were purchased from Ozyme (Saint-Quentin-en-Yvelines, France). For the variation in intron 17, for which no restriction site could be modified by the variation, common and variant homozygous samples were discriminated by dHPLC of a mixture of a known common homozygous sample with the unknown sample.

### Relationships between gene polymorphisms and drug cytotoxicity

After identification of the genotypes of each cell line, the IC_50_ values of 138 core drugs *vis-à-vis* the 59 cell lines, expressed as –log_10_(IC_50_), were extracted from the NCI database; mean values were calculated for common homozygous, variant homozygous and heterozygous cell lines for each of the variations encountered, and were compared by analysis of variance. Drugs were then grouped as a function of their known mechanism of action into eight categories (see Scherf *et al* (2000) for details): alkylating or platinating agents acting on N^7^ of guanine; other alkylating agents, acting on N^2^ and O^6^ of guanine; antimetabolites; antifolates; topoisomerase I inhibitors; topoisomerase II inhibitors; spindle poisons, which could be subdivided into *vinca*-alkaloid-type and taxane-type mechanisms of action. Only 10 drugs out of 138 remained unclassified because of disagreement about their precise mechanism of action. In order to compare drug groups, the individual IC_50_ values were normalised by subtracting individual values from the mean value of the 60-cell line panel. Using a univariate general linear model, we compared the variance of the IC_50_s for each genotype and we calculated the significance of the differences in the mean IC_50_s of each drug group as a function of the genotype of the cell lines. This model allows to take into account the unbalanced size of the groups. Due to the number of tests performed, we considered as significant only the *P*-values lower than 2.5 × 10^−4^ (Bonferroni correction). All analyses were performed with the SPSS software (version 12.0).

## RESULTS

### Research of exonic SNPs

We studied by dHPLC the PCR products originating from the 21 exons explored, including the intron–exon junctions, and from the 5′ untranscribed region up to 350 nucleotides before exon 1, using the primers indicated in Table 1. The presence of heteroduplexes was detected in only four PCR products over more than 2000 dHPLC runs that have been performed: in exon 13 of the IGROV1 cell line (ovarian cancer), in exon 17 of the MOLT-4 (leukaemia) and KM-12 (colon cancer) cell lines, and in exon 19 of the HCT-15 line (colon cancer). We explored five exons in the control population (exons 9, 12, 15, 19 and 21) that were selected because they contained the variations encountered in cell lines selected with camptothecin. No heteroduplex was detected in any of these five exons.

After sequencing, it appeared that three of the four variations detected in the cell lines of the NCI panel were indeed intronic, at a short distance of the exon–intron junctions, but not involving the splice site. The only true exonic variation observed was the one located in exon 19 of the HCT-15 cell line; it consists of a G>A transition at position 75 of the exon, resulting in a met>ile amino-acid change at position 675 of the protein.

Because of the very low frequency of the heterozygous DNAs, we found it unlikely to expect the presence of corresponding homozygous variants in either the control population or the NCI panel and no systematic sequencing of the PCR products was undertaken.

### Research of intronic SNPs

We first studied by dHPLC the PCR products originating from the five introns explored, at the sites where a SNP had been mentioned in databases with a frequency >0.25, using the primers indicated in Table 2. There were numerous heterozygous samples in the NCI panel. We verified, by sequencing 10 randomly selected samples for each intron, that each variation observed was the one expected from databases. We then set up RFLP techniques for the rapid identification of homozygous (common and variant) samples for each of the variations, as well as a confirmation for the heterozygous samples. Table 3 presents the distribution of the intronic variations among the 59 cell lines of the panel. The frequency of the variations detected was between 20 and 40%.

It clearly appeared that there was a strong linkage disequilibrium between the intronic variations studied. A total of 10 different genotypes were identified and, from these data, it was possible to identify three major haplotypes and four minor haplotypes. A major haplotype (60%) was characterised by the absence of all the intronic variations studied, the second one (16%) by the presence of the variations in introns 3, 6 and 17, and the third one (20%) by the presence of all the intronic variations studied. We propose to name these haplotypes A, B and C, respectively. The minor haplotypes were each present in only one cell line and contained 1–4 of the intronic variations studied. The combinations of the three major haplotypes generated 92% of all diplotypes and only four cell lines contained another haplotype.

### Relationships between gene polymorphisms and drug cytotoxicity

The HTC-15 cell line (colon cancer) was the only one to harbour a non-synonymous SNP, located in exon 19. The –log(IC_50_) of camptothecin in this cell line was 6.82, as mentioned in the NCI database, to be compared with the mean value (±s.d.) of camptothecin –log(IC_50_) in the 60 cell lines of the panel (7.38±0.55). This corresponds to a 3.6-fold resistance of the HCT-15 cell line as compared to the average cell line. Similar findings were obtained with the other topoisomerase I inhibitors of the camptothecin family (topotecan, SN-38). The –log(IC_50_) of rebeccamycin, the indolocarbazole lead compound tested against the NCI panel, is 6.40 in the HCT-15 cell line, which is among the five least sensitive cell lines of the whole panel to this agent (mean value for the panel: 6.70±0.26). It is not possible, however, to assign a causal relationship between this relative low sensitivity and exon 19 polymorphism, because the HTC-15 line appears as globally chemoresistant to most anticancer drugs (Scherf *et al*, 2000).

We compared the mean IC_50_s of 138 core drugs, extracted from the NCI database, in the three genetic status (wild-type homozygous, heterozygous, variant homozygous) of each of the intronic variation studied. Because of the small number of cell lines (3) exhibiting intron 2 or intron 8 variations at the homozygous state, we excluded them from the comparative study because of the possibility of bias. Comparison was also made as a function of the presence or absence of each of the three main haplotypes in the genotype of the cell lines. When considered individually, none of the 138 drugs had IC_50_ values significantly different according to the genotype, after Bonferroni adjustment. We then constituted drug groups on the basis of their mechanism of action (Scherf *et al*, 2000), and compared the normalised mean IC_50_ values of each drug group in the different genotypes. In order to avoid to take into account apparently significant relationships between drug sensitivity and polymorphic variations, which would result from chance only, we exclusively considered as relevant the differences in mean IC_50_ values of at least 50% (IC_50_ ratios >1.5 or <0.67) (biological significance) and giving a *P*-value <10^−4^ (statistical significance). The presence of variations in introns 2 and 8 (which are concomitant in 21 cell lines) appeared associated to a higher sensitivity to antifolates and taxanes (Table 4), whereas the presence of variations in introns 3, 6 and 17 (which are concomitant in 33 cell lines) was associated to sensitivity to topoisomerase I inhibitors and to resistance to taxanes.

**Figure 2:**
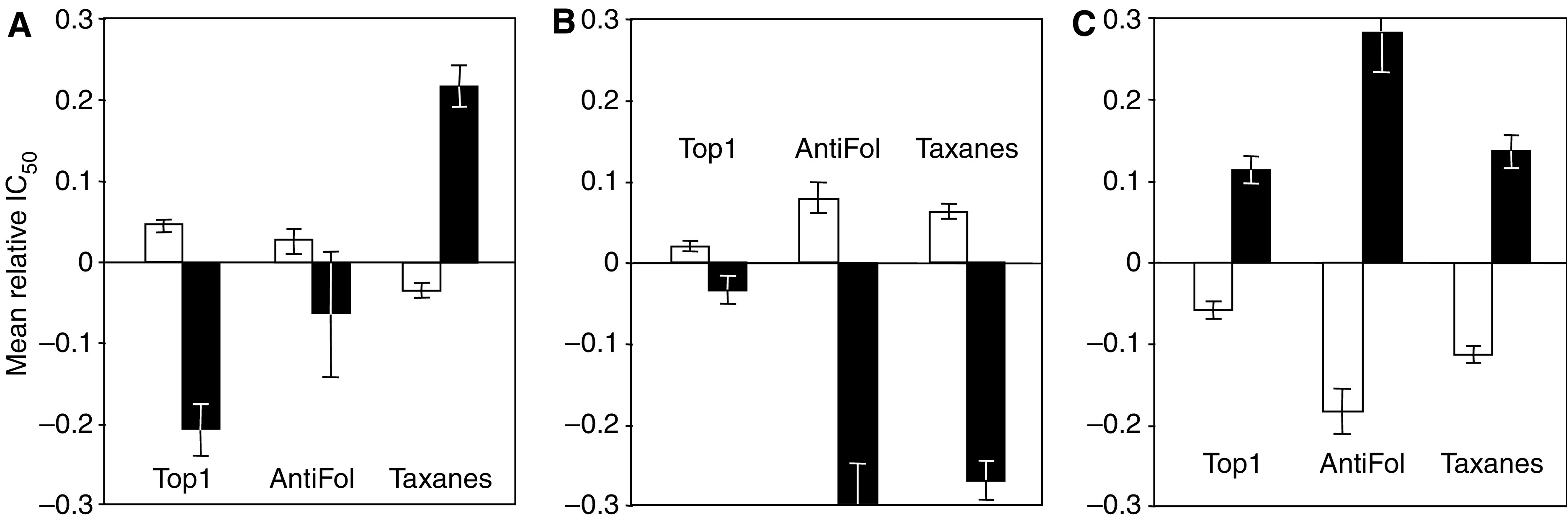
Schematic representation of the association between the presence or absence of a given allele in the genotype of the cell lines of the NCI-60 panel and the cytotoxicity of Top 1 inhibitors, antifolates and taxanes. The mean IC_50_s of drug classes towards the 60 cell lines, expressed as –log(IC_50_), were equalled to zero, the cytotoxicities higher than the mean were given a positive sign and the cytotoxicities lower than the mean a negative sign. White columns: absence of allele A, B or C in the genotype; black columns: presence of allele A, B or C in the genotype.

When haplotypes were considered, it appeared that the presence of at least one allele A in the genotype (47 cell lines) was significantly associated to resistance to Top1 inhibitors and to sensitivity to taxanes; the presence of at least one allele B (14 cell lines) was significantly associated to sensitivity to taxanes and antifolates; and the presence of at least one allele C (22 cell lines) was associated to sensitivity to Top 1 inhibitors, antifolates and taxanes (Figure 2).

We also compared the Top1 expression data, as extracted from the NCI database, to the genetic status of the intronic variations and of haplotype distribution. Among the various microarray data in the NCI database (http://dtp.nci.nih.gov), those performed independently by two groups with the U95A and the U133A Affymetrix chips appeared as the more reliable and they were used for comparisons with drug cytotoxicity and with *TOP1* genotypes. There was no significant correlation between *TOP1* gene expression and the cytotoxicity of Top1 inhibitors *vis-à-vis* the NCI-60 panel, and we found no correlations either between *TOP1* gene expression and the gene polymorphisms. As the *TOP1* gene copy number could be a confounding factor for establishing relationships between gene polymorphisms and gene expression, we corrected the expression data in the NCI database by dividing the expression levels by the number of chromosome 20q arms in the cell line, as extracted from the karyotype description (http://www.ncbi.nlm.nih.gov/sky/skyweb/cgi), but we evidenced not better relationships.

## DISCUSSION

This is the first time, to our knowledge, that the genomic variations of Top1 were studied in a variety of tumour and normal samples. Topoisomerase 1 appears in this study as a highly conserved protein, with exceptional variations in the coding sequence. This is probably in relation to the crucial importance of this enzyme activity in cell life. Only one exonic variation was found in 59 different tumour cell lines and 53 DNA samples of healthy individuals. This variation is able to determine an amino-acid change at position 675 of the protein, in a domain which is not far from the catalytic tyrosine (amino acid 723) but in a linker region where no drug-induced mutation able to confer drug resistance has been identified (Ohashi *et al*, 1996; Takatani *et al*, 1997; Pommier *et al*, 1999; Tsurutani *et al*, 2002; Woo *et al*, 2002). For this reason, we have not tried to detect the variant protein in the corresponding cell line and we do not know whether it is expressed and whether it shares the same catalytic properties as the common enzyme. It is also difficult to know whether this variation is a polymorphism that can be encountered in normal subjects with low frequency or a tumour somatic mutation generated during the process of oncogenesis.

This is also the first report on the haplotype distribution of *TOP1* in a panel of human tumours. The most frequent intronic variations described in the databases have been found at the expected location and with the expected frequency. As we have explored, from intron 2 to intron 17, about 82% of the length of the *TOP1* gene, we have obtained a global insight of the haplotype distribution. Only three different haplotypes were present in 55 cell lines out of 59, the four remaining cell lines harbouring different haplotypes, in combination with the A and C haplotypes. Haplotypes were distributed randomly among the various cell lines, with no specificity for the tissue of origin of the tumour cell lines, suggesting that they reflect the constitutive distribution of the individuals from whom the tumour was isolated and grown in culture. There was no relationship between haplotype distribution and the cytogenetic status of the cell lines, which was studied in detail by Roschke *et al* (2003) and is freely available on the NCBI site (http://www.ncbi.nlm.nih.gov/sky/skyweb/cgi): the three main haplotypes were present in cells which were diploid, polyploid or aneuploid for chromosome 20, and so were also the rare haplotypes.

The intronic SNPs of *TOP1* have been determined by the Hapmap consortium in a normal Caucasian population of 91 subjects from the Centre d'Étude du Polymorphisme Humain (CEPH) (http://www.hapmap.org), 84 of whom could be assigned as presenting a combination of the three main haplotypes A, B and C. The frequencies of alleles A, B and C were similar in this population and in the NCI panel. However, when considering the distributions of A, B and C haplotypes in terms of common homozygous, heterozygous and variant homozygous genotypes, it appeared that the number of AB heterozygous samples was slightly lower than expected in the NCI panel from the Hardy–Weinberg distribution (*P*=0.073). This means that a loss of heterozygosity may have occurred during the process of oncogenesis for allele B, but not for allele C. As a consequence, several cell lines considered as homozygous for allele B must rather be hemizygous for at least a part of chromosome 20. Loss of heterozygosity is also evident from the comparisons that can be done on cell lines which have been recently shown to originate from the same individual (Holbeck, personal communication, August 8, 2005; see Garraway *et al*, 2005): NCI/ADR-RES and OVCAR-8, M14 and MDA-MB-231 and U251 and SNB-19. For two of these pairs, the genotypes we identified were different, which can only be explained by loss of heterozygosity having occurred in one of the cell lines.

The relationships between drug cytotoxicity and the polymorphism of *TOP1* cannot be explained by a simple effect on *TOP1* gene expression or enzyme intrinsic activity, as no significant relationship could be evidenced between the polymorphisms and the gene expression data, even after correction by the *TOP1* gene copy number. The gene expression database of the NCI may not appear very robust, with 10 microarray data sets obtained with various distinct oligonucleotides, and no data set obtained with a reference technique such as quantitative RT–PCR. We only used the microarray data obtained with the U95A and the U133A Affymetrix chips, which were provided by two independent groups working on distinct RNA extracts and gave strongly correlated results. There appears to be a lower reliability of data from the U95B-E and U133B chips, which has been observed with many other genes and may reflect incorrect mapping of the gene to that Affymetrix feature. Even if we missed a correlation between *TOP1* polymorphisms and Top1 expression, the mechanism by which the corresponding haplotype would influence Top1 expression remains elusive. Furthermore, the relationships between cell sensitivity to antifolates and taxanes and the haplotype distribution of *TOP1* genotypes cannot be explained at this point from a pharmacological point of view.

It must be kept in mind that polymorphisms may only be the reflect of unidentified variations, occurring at a distance from the SNP studied, and playing the actual mechanistic effect on the cellular properties observed. Several genes involved in cell proliferation and oncogenesis are present in the vicinity of the *TOP1* gene and belong to the same haplotypic block: they could well be the true responsible for the association observed between *TOP1* polymorphisms and drug cytotoxicity. This is especially the case for the gene *ZHX3*, which encodes a zinc-finger protein involved in the repression of transcription. There is a common non-synonymous polymorphism on this gene (Ser310Asn), with a rare allele frequency of 0.208 in Caucasians, which is significantly associated with the variant haplotypes of the *TOP1* gene, and whose functionality is not known and should be explored (http://www.ncbi.nlm.nih.gov/SNP/snp_ref.cgi?rs=17265513).

Nevertheless, even if the mechanism relating *TOP1* polymorphisms to drug cytotoxicity remains unidentified, it might be of interest to identify such associations, that may be used as predictive factors of drug activity, once the *in vitro* observation has been transferred to the clinical setting. We are planning retrospective studies on DNA samples from cancer patients who have been treated, for instance, with combinations of antifolates (such as raltitrexed) and Top1 inhibitors (such as irinotecan) in order to validate our observations.

## Figures and Tables

**Table 1:** Primer sequences for the PCR of the 21 exons of the *TOP1* gene

**SNP**	**Localisation^a^**	**Primer sequence**	**Exon length (bp)**	**Amplicon length (bp)**
5′ UTR	G4709344T rs6072250	S: GATACCAGCTCTGCCCAAGA As: AGACTCCAGAAACGGCTGAG	—	656
Exon 1		S: CCGACGTGTTGTTTAAAAG As: CCTAGCGAGCCGATCTAC	279	474
Exon 2		S: GGCCGCGAAGTTACAGTT As: ACGCCATGTCTGTCCTCT	25	285
Exon 3		S: TGTCAAGGTAGGCATACAGA As: GCTGAATGATGAGGGACTT	97	201
Exon 4		S: GGCTAACGCTTTGTGACTTA As: CCTTCCTCCCCTACTAGAAA	124	184
Exon 5		S: CCAGGAACTGATGCTACATA As: TTGGGTTAACTCAGACAACA	56	233
Exon 6		S: CACTCTGACCAGCAATTTTT As: AGTTCAGAGAAATCAGTCCA	96	280
Exon 7		S: TGTATTCATGTTCCCCTTTC As: AGCCCCAAAGGACCTAAG	76	130
Exon 8		S: GCTTTCTCACCATGTTTCTT As: ATTTTAACCCAGTGCTGAGA	107	158
Exon 9	G4774052A (Gly214Ser) rs6029542	S: ATCACTAAATGAGGCTGTGC As: TCTATTACATAGGATCCTTCCTT	116	242
Exon 10		S: TCACTTTTTGGAACCACTTT As: ACTTGATTGTTTTGCAAGGT	122	201
Exon 11		S: TCCCCATTTTCTTTTGACTA As: ATTCCAGAATTCTCCAGAGG	123	184
Exon 12	G4781682A (Arg349Arg) rs6029545	S: TCAGACTTTCCTCTACCTTGA As: CTTTTTGGCCCATAGGAT	188	325
Exon 13	Gly418Lys	S: TTTCACTATCCTCGTGCTCT As: AGGTTATCAGCACCATGAAC	145	223
Exon 14		S: TGCCTGGCTATATTCAAAGT As: GATGGGAAGATGCTCTCAC	144	238
Exon 15	G4795621A (Glu517Lys) rs6129757	S: ATGTCTCTTCCATTCATGCT As: ACCCATGCTTGTTGAATAAT	186	288
Exon 16		S: TGCAAGTTTCTTTAAGTTTGG As: TTCAACATTGTGTGCTTCAT	69	181
Exon 17	G4797898A (Thr591Thr) rs1061982	S: TTCCTCTTTCCCTAACTTCC As: ATCTGGCCAAGCAATACTTA	115	276
Exon 18		S: GATAAGAGCCAGCAAAATCA As: TCCTCTCATTCGCAGATAAT	128	300
Exon 19	T4803328C (Lys676Lys)	S: CATCTTGGTTTCACCTTCTC As: GCTGCACACTTTTCTCTACC	95	222
Exon 20	A4803680G (Asn722Ser)	S: GGTTCTTGAGGACTTTGCTA As: AAGGAGGGCTCAACACTTAC	150	199
Exon 21	G4804749A (splice site) rs6072277	S: CATTGCTGAGTCACCCTAAT As: AGTGAGGCTCAGTTTATCCA	107	224
	G4804829C (Met758Ile) rs12057885			

a This refers to mutations or SNPs mentioned in databases and that have been especially sought.

**Table 2:** Primer sequences for the PCR of the five intronic regions known as harbouring frequent SNPs

**SNP**	**Localisation^a^**	**Restriction enzyme**	**Primer sequence**	**Intron length (bp)**	**PCR product length (bp)**
Intron 2	T4717580C rs1265035	*Bfa*I	S: GTAAGGTAGCCCCTTTGTTT As: CTTTCCTGGTCTCTGGTTCT	31 938	185
Intron 3	T4743257C rs1997833	*Mse*I	S: TTAGGCCTTAAATGGGATG As: CAAGCTTGCTACAGACTTGA	14 680	163
Intron 6	T4762170C rs6016511	*Mnl*I	S: TCCTGTCAGACATTCTAAGTGA As: CCTTTCCATCCAATTTCTACT	984	177
Intron 8	C4768132T rs6124314	*Hin*P1I	S: GTGGTAGTTACTCAATAAATGCT As: AGGGCACCTAATTCTACACA	7903	156
Intron 17	A4799318G rs6129760	—	S: TCAATTCCTCGTTCTGTTCT As: TTCATCTCCTTGCTTCCTC	1776	152

a This refers to the variations mentioned in databases.

**Table 3:** Distribution of the intronic SNPs among the cell lines of the NCI-60 panel

**Tumour type**	**Cell line**	**Intron 2 rs1265035**	**Intron 3 rs1997833**	**Intron 6 rs6016511**	**Intron 8 rs6124314**	**Intron 17 rs6129760**	**Diplotype^a^**
Leukaemia	CCRF-CEM	HT	V	V	HT	V	BC
	HL-60	W	W	W	W	W	AA
	K-562	HT	HT	HT	HT	HT	AC
	MOLT-4	W	W	W	W	W	AA
	RPMI-8226	HT	HT	V	HT	V	
	SR	HT	V	V	V	V	

Lung cancer	A549/ATCC	W	W	W	W	W	AA
	EKVX	HT	V	V	HT	V	BC
	HOP-62	V	V	V	V	V	CC
	HOP-92	HT	HT	HT	HT	HT	AC
	NCI-H226	HT	HT	HT	HT	HT	AC
	NCI-H23	W	HT	HT	W	HT	AB
	NCI-H322M	W	W	W	W	W	AA
	NCI-H460	W	W	W	W	W	AA
	NCI-H522	W	W	W	W	W	AA

Colon cancer	COLO-205	W	W	W	W	W	AA
	HCC-2998	W	W	W	W	W	AA
	HCT-116	W	V	V	W	V	BB
	HCT-15	W	W	W	W	W	AA
	HT29	W	HT	HT	W	HT	AB
	KM12	W	W	W	W	W	AA
	SW-620	HT	HT	HT	HT	HT	AC

Central nervous system	SF-268	W	W	W	W	W	AA
	SF-295	W	HT	HT	W	HT	AB
	SF-539	W	W	W	W	W	AA
	SNB-19	W	V	V	W	V	BB
	SNB-75	W	W	W	W	W	AA
	U251	W	W	W	W	W	AA

Melanoma	LOXIMVI	V	HT	V	HT	V	
	MALME-3M	W	HT	HT	W	HT	AB
	M14	HT	HT	HT	HT	HT	AC
	SK-MEL-2	W	W	W	W	W	AA
	SK-MEL-28	HT	HT	W	W	W	
	SK-MEL-5	HT	HT	HT	HT	HT	AC
	UACC-257	W	HT	HT	W	HT	AB
	UACC-62	HT	HT	HT	HT	HT	AC

Ovarian cancer	IGROV1	HT	HT	HT	HT	HT	AC
	OVCAR-3	W	W	W	W	W	AA
	OVCAR-4	W	V	V	W	V	BB
	OVCAR-5	W	HT	HT	W	HT	AB
	OVCAR-8	W	W	W	W	W	AA
	SK-OV-3	V	V	V	V	V	BB

Renal cancer	786-0	HT	HT	HT	HT	HT	AC
	A498	HT	HT	HT	HT	HT	AC
	ACHN	V	V	V	V	V	CC
	CAKI-1	W	W	W	W	W	AA
	RXF-393	W	W	W	W	W	AA
	SN-12C	HT	HT	HT	HT	HT	AC
	TK-10	W	W	W	W	W	AA
	UO-31	W	V	V	W	V	BB

Prostate cancer	PC-3	HT	HT	HT	HT	HT	AC
	DU-145	W	W	W	W	W	AA

Breast cancer	MCF-7	HT	HT	HT	HT	HT	AC
	NCI/ADR-RES	W	W	W	W	W	AA
	MDA-MB-231	W	W	W	W	W	AA
	HS578T	HT	HT	HT	HT	HT	AC
	MDA-MB-435	HT	HT	HT	HT	HT	AC
	BT-549	W	W	W	W	W	AA
	T-47D	W	HT	HT	W	HT	AB

a Four diplotypes could not be ascertained due to the presence of rare haplotypes.

**Table 4:** Relationships between intronic SNPs and drug sensitivity in the cell lines of the NCI-60 panel

		**Top1 inhibitors (18)**		**Antifolates (11)**		**Taxanes (13)**	
		**IC_50_ ratio**	***P*-value**	**IC_50_ ratio**	***P*-value**	**IC_50_ ratio**	***P*-value**
Intron 2^a^	HT *vs* W	1.16	0.022	2.22	8.0 × 10^−7^	1.90	1.9 × 10^−11^
Intron 8^a^	HT *vs* W	1.25	6.8 × 10^−5^	2.69	5.7 × 10^−8^	2.12	1.5 × 10^−12^
Intron 3	V *vs* W	1.85	5.7 × 10^−8^	1.39	0.86	0.46	2.8 × 10^−9^
	V *vs* HT	1.83	1.1 × 10^−7^	0.79	0.037	0.34	2.1 × 10^−11^
Introns 6 and 17^b^	V *vs* W	2.01	1.0 × 10^−8^	2.21	0.064	0.69	2.3 × 10^−5^
	V *vs* HT	1.84	5.3 × 10^−7^	1.32	0.930	0.50	1.5 × 10^−8^
Allele A	Pres. *vs* Abs.	1.91	6.0 × 10^−9^	1.69	0.272	0.58	1.8 × 10^−9^
Allele B	Abs. *vs* Pres.	1.16	0.006	2.72	8.1 × 10^−7^	2.21	2.6 × 10^−12^
Allele C	Abs. *vs* Pres.	0.70	5.0 × 10^−10^	0.39	4.4 × 10^−8^	0.58	1.0 × 10^−10^

Abbreviations: Abs., absent; HT, heterozygous; Pres., present; V, variant; W: wild-type.

a Due to the small number (three) of homozygous variants for these variations, comparisons of their IC_50_s with wild-type and heterozygous samples were not made.

b All cell lines presented the same variations of introns 6 and 17.
